# Deciphering the virome of Chunkung (*Cnidium officinale)* showing dwarfism-like symptoms via a high-throughput sequencing analysis

**DOI:** 10.1186/s12985-024-02361-7

**Published:** 2024-04-15

**Authors:** Mesele Tilahun Belete, Se Eun Kim, Workitu Firmosa Gudeta, Davaajargal Igori, Jeong A. Kwon, Su-Heon Lee, Jae Sun Moon

**Affiliations:** 1 Biosystem and Bioengineering Program, University of Science and Technology (UST), Daejeon, 34141 Republic of Korea; 2 Plant System Engineering Research Center, Korean Research Institute of Bioscience and Biotechnology, Daejeon, 34141 Republic of Korea; 3 Amhara Agricultural Research Institute, Plant Biotechnology Research Division, Bahir Dar, Ethiopia; 4 Department of Biology, School of Mathematics and Natural Sciences, Mongolian National University of Education, Ulaanbaatar, Mongolia; 5 School of Applied Bioscience, College of Agriculture and Life Sciences, Kyungpook National University, Daegu, 98411 Republic of Korea

**Keywords:** Plant virus, Viral diversity, Virome, *Cnidium officinale*, Proximity sampling, HTS, Dwarfed

## Abstract

**Background:**

Viruses have notable effects on agroecosystems, wherein they can adversely affect plant health and cause problems (e.g., increased biosecurity risks and economic losses). However, our knowledge of their diversity and interactions with specific host plants in ecosystems remains limited. To enhance our understanding of the roles that viruses play in agroecosystems, comprehensive analyses of the viromes of a wide range of plants are essential. High-throughput sequencing (HTS) techniques are useful for conducting impartial and unbiased investigations of plant viromes, ultimately forming a basis for generating further biological and ecological insights. This study was conducted to thoroughly characterize the viral community dynamics in individual plants.

**Results:**

An HTS-based virome analysis in conjunction with proximity sampling and a tripartite network analysis were performed to investigate the viral diversity in chunkung (*Cnidium officinale*) plants. We identified 61 distinct chunkung plant-associated viruses (27 DNA and 34 RNA viruses) from 21 known genera and 6 unclassified genera in 14 known viral families. Notably, 12 persistent viruses (7 DNA and 5 RNA viruses) were exclusive to dwarfed chunkung plants. The detection of viruses from the families *Partitiviridae*, *Picobirnaviridae*, and *Spinareoviridae* only in the dwarfed plants suggested that they may contribute to the observed dwarfism. The co-infection of chunkung by multiple viruses is indicative of a dynamic and interactive viral ecosystem with significant sequence variability and evidence of recombination.

**Conclusions:**

We revealed the viral community involved in chunkung. Our findings suggest that chunkung serves as a significant reservoir for a variety of plant viruses. Moreover, the co-infection rate of individual plants was unexpectedly high. Future research will need to elucidate the mechanisms enabling several dozen viruses to co-exist in chunkung. Nevertheless, the important insights into the chunkung virome generated in this study may be relevant to developing effective plant viral disease management and control strategies.

**Supplementary Information:**

The online version contains supplementary material available at 10.1186/s12985-024-02361-7.

## Background

*Cnidium officinale* (Korean name: chunkung), which is a flowering annual plant in the family Apiaceae [1, 2], has a long history of use in traditional medicine in Far East Asia, especially in Korea, China, and Japan. It has been used to treat various conditions, including pruritus, skin disorders, asthma, and erectile dysfunction [2–6]. Chunkung plants are cultivated through their rhizomes and are used to relieve pain and treat menstrual disturbances [7], vitamin deficiencies [8], hypertension, and inflammation [9]. Active compounds, such as osthol [5], volatile alkyl phthalide derivatives, and polysaccharides, have also been identified in chunkung rhizomes. To date, 350 compounds have been isolated from chunkung and identified [5]. There are ongoing studies on the medicinal properties of chunkung as well as on the phytopathogens (e.g., viruses) that can infect chunkung [10].

Viruses, which are ubiquitous in the biosphere, can infect a wide range of hosts, including plants, animals, and microbes [11]. They can invade their hosts through a process called cross-species transmission or spillover [12–14], which can adversely affect plant health, thereby decreasing the market value of cultivated plants [15–19]. Viral species in agricultural systems differ significantly from those in natural ecosystems [12]. In the latter case, including wild plants and native vegetation, have a distinct relationship with their host plants and often have neutral effects or provide their hosts with slight advantages [20]. In agricultural systems, they can significantly affect plant growth, resulting in substantial yield losses [21–23]. Plant viruses are naturally transmitted through a variety of pathways (e.g., pollen, seeds, and vectors) [24, 25]. High-density cultivation of plants can promote viral spread [26]. The diversity of viruses in ecosystems can be influenced by various factors [27]. These include changes in the genetic traits and composition of host populations, shifts in the ecology of both the host plant and the virus, and for vectored viruses, modifications in the vector’s ecology and genetics. Additionally, human practices also affect viral diversity. For example, monocultures, irrigation systems, extended growing seasons, the transport of seedlings, changes in land use, and the application of artificial soil amendments can alter ecological dynamics and favor the emergence and spread of specific plant viruses [20, 27]. Several economically important viral diseases affect chunkung [28, 29], and the emergence of novel viruses is increasingly being detected because of advances in high-throughput sequencing (HTS) technologies.

Predicting crop damage is difficult [30] due to multiple factors, such as geographic region, virus strain, host plant cultivar/variety, and timing of infection [19, 31]. Technological advances, surveillance efforts, and improved diagnostic capabilities have increased interest in plant virus research [32, 33]. The development of massively parallel sequencing, which is also known as HTS, has contributed to significant breakthroughs in virome research [20, 25, 30, 34, 35]. The diagnosis of viral diseases typically relies on specific techniques (e.g., ELISA, microarray, and PCR) [34, 36] that are relatively cheap/economic and can rapidly detect known viruses [37]. However, these methods may be inappropriate for examining samples co-infected with multiple viral agents [38, 39]. Alternatively, HTS can efficiently detect and identify multiple viruses, even in the absence of prior knowledge about viral sequences [21, 26]. Advances in nucleic acid isolation protocols and the availability of HTS technologies have enabled researchers to comprehensively study the viruses associated with a specific host [39, 40]. Until recently, our understanding of plant virus diversity was limited. However, over the past decade, the mainstream use of HTS in biodiversity surveys, has helped fill our knowledge gaps. As a result, full viromes [16] obtained by HTS approach may lead to significant breakthroughs by enabling inclusive viral genome analyses, facilitating metagenomic investigations [41, 42], expanding analyses to non-model hosts [43], identifying viral variants [16], and advancing our understanding of viral transcriptomics [32].

The objective of this study was to elucidate the viral diversity in chunkung via HTS. We conducted a comprehensive survey to identify viruses that may be responsible for putative growth abnormalities and complex infections, or that adversely affect chunkung plant growth. The results of this study offer valuable insights into the diversity of viral communities and their potential ecological interactions. Furthermore, the virus genomes that were annotated and classified in this study are valuable resources for future research on virus–host interactions and the development of disease management strategies applicable to chunkung (SRA accessions: SAMN39861745 and SAMN39861546 of the BioProject PRJNA1074493). To the best of our knowledge, this survey represents the first study of the chunkung virome.

## Methods

### Sample collection and processing

**Fig. 1:**
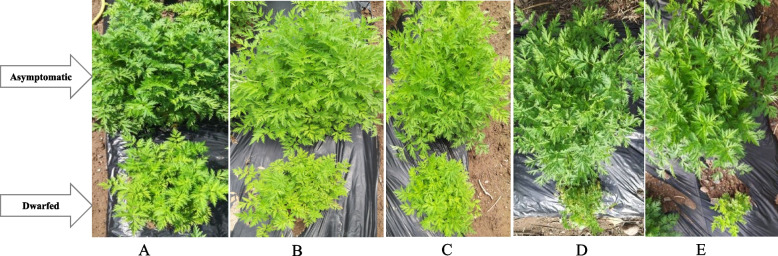
Samples of chunkung were collected for virome analysis. Panels **A**–**E** depict five sets of duplicate ChunKung plants. Each pair of images contrasts different growth characteristics: the upper group shows asymptomatic plants, characterized by larger morphology and higher yields, while the bottom group shows dwarfed plants exhibiting abnormal growth, stunted development, and unproductive features

We collected five duplicates of chunkung plants from three farms in Yeongyang, Gyeongsangbuk-do Province, South Korea (Table 1), which has conditions suitable for chunkung cultivation. All chunkung samples were cultivated in the field. In June 2021, five asymptomatic plants (designated as A) and five dwarfed plants (designated as D) were collected (Fig. 1A-E, Table 1). The strategy of collecting paired plant samples based on their proximity (closeness) was employed to minimize potential biases that could arise from variations in soil texture, fertility, and other environmental factors. More specifically, dwarfed and asymptomatic plants growing in close proximity were collected (Fig. 1A-E). Each sample was finely ground using a mortar and pestle precooled with liquid nitrogen. The powdered materials were stored at − 80 °C until further processing. For HTS, 4 g of each sample was pooled into two groups (i.e., asymptomatic and dwarfed), which were used to generate two libraries (i.e., A and D). Pooling samples can reduce sequencing costs and increase the chances of identifying multiple viruses simultaneously. However, it limits the detailed virome information from individual plants. Before RNA-Seq, we confirmed known virus infections using RT-PCR, allowing us to compare viromes from each sample.

**Table 1:** List of *Cnidium officinale* leaf samples: five dwarfed and five asymptomatic plants collected from Gyeongsangbuk-do province in Korea for RNA-seq

No	Sample name	Sample code	Collection date	Collection place
1	*Cnidium officinale* 1 (dwarfed)	1D	2021.06.12	236–4 Dogok-ri, Ilwol-myeon
	*Cnidium officinale* cryptic (Asymptomatic) 1	1A	2021.06.12	236–4 Dogok-ri, Ilwol-myeon
2	*Cnidium officinale* 2 (dwarfed)	2D	2021.06.12	236–5 Dogok-ri, Ilwol-myeon
	*Cnidium officinale* cryptic (Asymptomatic) 2	2A	2021.06.12	236–5 Dogok-ri, Ilwol-myeon
3	*Cnidium officinale* 3 (dwarfed)	3D	2021.06.12	480–9 Seomchon-ri, Ilwol-myeon
	*Cnidium officinale* cryptic (Asymptomatic) 3	3A	2021.06.12	480–9 Seomchon-ri, Ilwol-myeon
4	*Cnidium officinale* 4 (dwarfed)	4D	2021.06.12	480–10 Seomchon-ri, Ilwol-myeon
	*Cnidium officinale* cryptic (Asymptomatic) 4	4A	2021.06.12	480–10 Seomchon-ri, Ilwol-myeon
5	*Cnidium officinale* 5 (dwarfed)	5D	2021.06.12	Gokgangri San 2
	*Cnidium officinale* cryptic (Asymptomatic) 5	5A	2021.06.12	Gokgangri San 2

### Total RNA extraction, RNA-seq library preparation, and sequencing

**Fig. 2:**
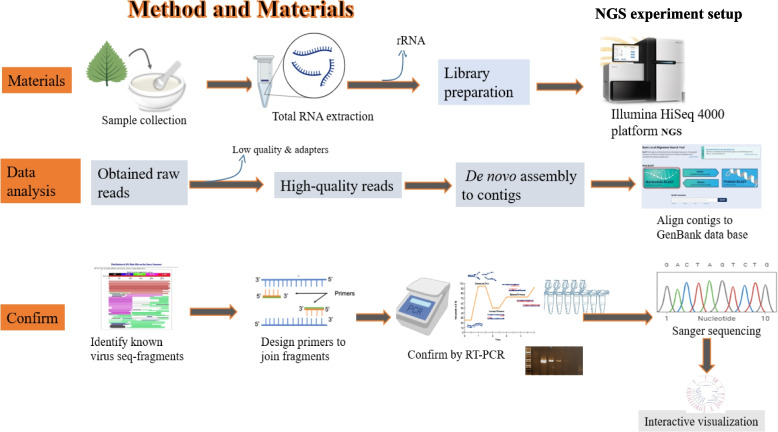
The virome analysis workflow for chunkung plants. Total RNA was extracted from each pooled sample. Libraries were prepared from the extracted RNA and sequenced using the Illumina HiSeq 4000 platform to generate the raw read data. Quality control was applied to obtain high-quality reads, and de novo assembly was used to reconstruct contigs from these reads. The contigs were aligned to the GenBank database for identification and comparison with known plant viral sequences. To confirm the presence of the viral species, primers were designed from the viral sequence fragments and validated by (RT-) PCR. Sanger sequencing was conducted to verify the obtained amplicons, and interactive visualization was performed. This comprehensive workflow involved sample collection, RNA extraction, library preparation, sequencing, data analysis, confirmation, and visualization for the virome analysis of chunkung plants

Total RNA was extracted from pooled asymptomatic and dwarfed samples as previously described [3, 4]. An easy-spin™ Total RNA Isolation Kit (iNtRON Biotechnology, Sangdaewon-dong, South Korea) was used to extract RNA according to the manufacturer’s protocol. The RNA quality, quantity, integrity were determined by gel electrophoresis and the Agilent 2100 Bioanalyzer (Agilent Technologies, CA, USA). The ribosomal RNA (rRNA) was eliminated using the Ribo-Zero™ rRNA Removal Kit (Plant Leaf) (Epicenter, Madison, WI, USA). Subsequently, libraries were generated using the TruSeq Stranded Total RNA low-throughput sample prep kit (Illumina, San Diego, CA, USA). The final sequencing libraries were then prepared for paired-end sequencing (2 × 101-bp reads), which was performed using the Illumina HiSeq 4000 system (Illumina, CA, USA) at Macrogen Co. (Seoul, South Korea) (Fig. 2).

### In silico analyses and assembly of viral genomes

The quality of the raw reads was assessed using FastQC (v0.11.7) [42]. Trimmomatic (v0.38) and the sliding window method [44] were used to remove including adapter sequences and low-quality reads (Phred < 30) [45]. The de novo assembly of the trimmed reads was performed using Trinity (vr20140717) [4]. The trimmed reads for each library were mapped to the assembled reference sequence (https://www.ncbi.nlm.nih.gov/genomes/GenomesGroup.cgi?taxid=10239; retrieved in July 2021) using Bowtie 1.1.2 [44]. To determine viral gene abundance in each library, the read counts were estimated and their expression was measured using RSEM (v1.2.29) [46]. For this analysis, multiple public databases (e.g., NCBI nucleotide, NCBI non-redundant protein, and Pfam databases) were searched to gather all-inclusive information. Transdecoder (v3.0.1) [47] was used to predict virus-specific open reading frames. The assembled contigs associated with the chunkung virome were characterized using DIAMOND (v0.9.21) [48] and the NCBI BLASTn and BLASTx algorithms. The results were filtered using the default E-value cutoff of 1.0E-5. We carefully examined these contigs to identify viruses (Additional file 1 Table S1), excluded those that had nonviral CD-search hits, and selected only those with reliable and significant Basic Local Alignment Search Tool (BLAST) hits.

### BLAST search for identifying viruses in chunkung

The viruses detected in the chunkung were identified on the basis of a BLAST search [49] (Additional file 1 Table S1). To identify viral species affecting chunkung plants, the assembled contigs were used as queries for BLAST analyses (E-value cutoff of 1.0E-5). The BLAST hits with significant similarities to known viruses were considered as potential chunkung viruses. Further analyses (e.g., virus taxa) were performed using iTOL (https://itol.embl.de/itol.cgi) [50]. Cytoscape (v3.10.0) [51], which is useful for visualizing and analyzing biological networks, was used to explore eventual virus–host interactions.

### Detection of chunkung viruses via (RT-) PCR assays

Primers were designed for the PCR amplification of specific target regions of the viruses identified according to the annotated viral contigs obtained by HTS. The PCR assay was completed to confirm the presence of the viruses in individual plant hosts (Fig. 1A-E.). The Primer3Plus tool (https://www.bioinformatics.nl/cgi-bin/primer3plus/primer3plus.cgi) [52] was used to design primers on the basis of the contigs corresponding to the identified species (Additional file 2 Table S8). To detect RNA viruses, total RNA was extracted from each chunkung sample using the WizPrep Plant Mini Kit (Wizbiosolutions, Seongnam, South Korea). Similarly, to detect DNA viruses, total DNA was extracted from each chunkung sample using the DNeasy Plant Mini Kit (Qiagen, Hilden, Germany). The RNA viruses were identified via a reverse transcription PCR (RT-PCR) performed using the one-step SuPrimeScript RT-PCR Premix (2 ×) (GeNet Bio, Daejeon, South Korea), whereas the DNA viruses were identified via a PCR performed using the Prime Taq Premix (GeNet Bio). The PCR products were analyzed by 1% agarose gel electrophoresis and ethidium bromide staining. The PCR products were purified using the HiYield™ Gel/PCR DNA Mini Kit (RBC Bioscience, Taipei, Taiwan), and sent to Macrogen (Seoul, South Korea) for Sanger sequencing. The obtained sequences were analyzed and trimmed using DNAMAN (v5.2.10) (Lynnon BioSoft, CA, USA) [5].

## Results

### Sample collection and analysis of the symptoms of virus-infected chunkung plants

Clear differences were detected between the asymptomatic and dwarfed plants. The upper group represents asymptomatic plants, which are distinguished by larger morphology and higher yields. In contrast, the bottom group represents dwarfed plants that grew abnormally (i.e., overall poor health, stunted development, and unproductive features) (Fig. 1A-E).

### Virome assembly and identification of viruses in chunkung

A total of 661,669,928 raw reads (66.8 Gbp) were obtained for the A library, whereas 655,644,488 raw reads (66.2 Gbp) were generated for the D library (Additional file 1 Table S2 2). For both libraries, the read length was 101 bp. Trimmomatic (v0.38) was used to remove barcode adapters and low-quality sequences. After trimming, 653,637,904 and 645,507,670 high-quality reads remained for the A and D libraries, respectively. The GC content was lower for the A library than for the D library (Additional file 1 Table S2). In addition, 529,312,518 and 344,279,682 trimmed reads (Additional file 1 Table S3) for the A and D libraries, respectively, were mapped to the assembled viral reference sequence using Bowtie. Moreover, 181,976 assembled transcripts with lengths ranging from 201 to 23,564 nucleotides were obtained for the A library, whereas 203,437 assembled transcripts with lengths ranging from 201 to 26,375 nucleotides were obtained for the D library (Additional file 1 Table S4). These transcripts were then compared with the sequences in various publicly available virus RefSeq databases.

**Fig. 3:**
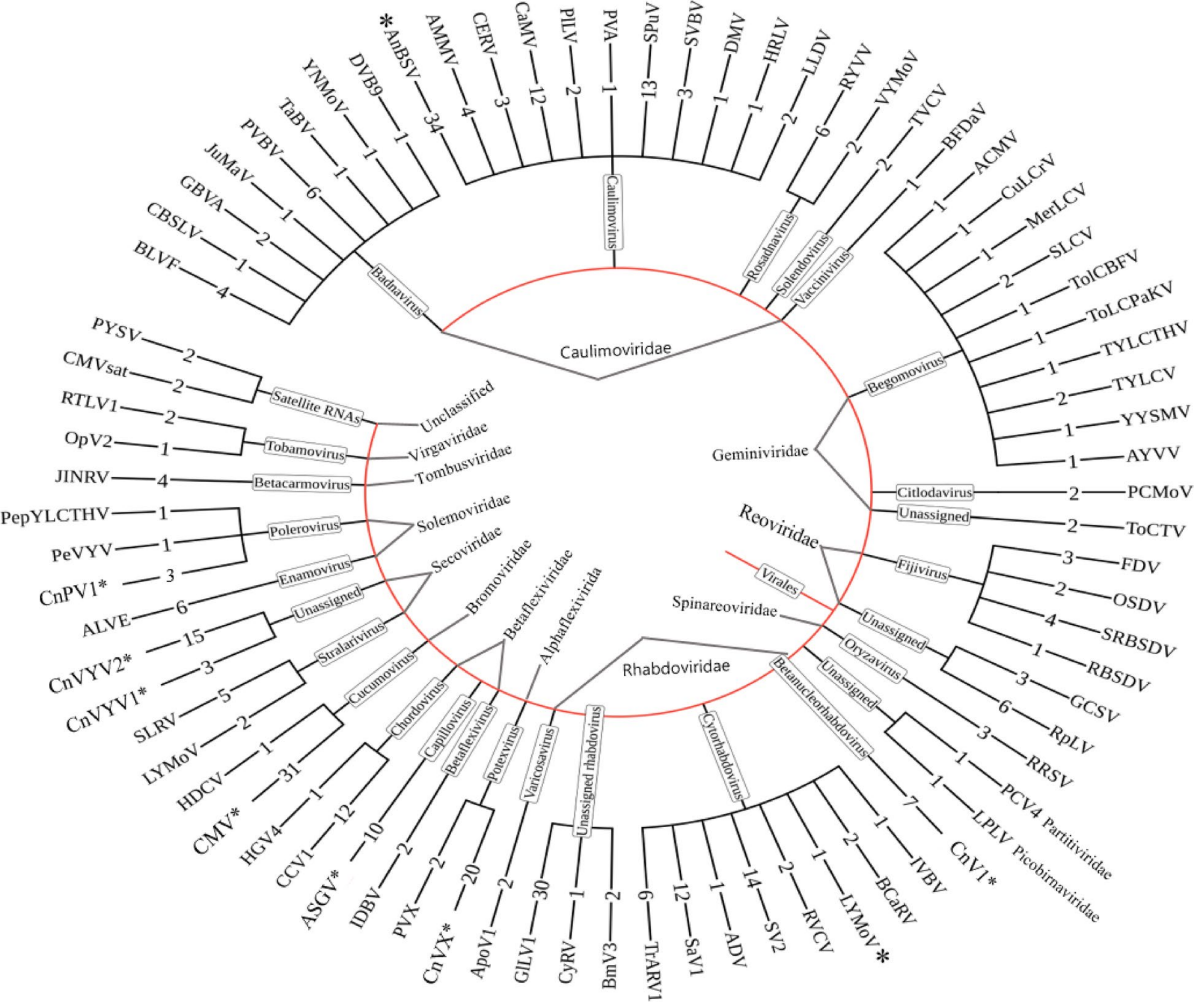
Comparative analysis of the virome of chunkung plants according to RNA-seq data. The Newick tree dendrogram was generated using iTOL. Genus names are displayed within rectangular boxes, with the corresponding families of each virus presented at the centers of the circles. The number of contigs corresponding to each viral species is denoted on the branch line preceding the virus’s acronym. viruses indicated with (*): viruses known to infect chunkugn plants in Korea and Japan. NB: All virus acronyms are described in Table 3

**Fig. 4:**
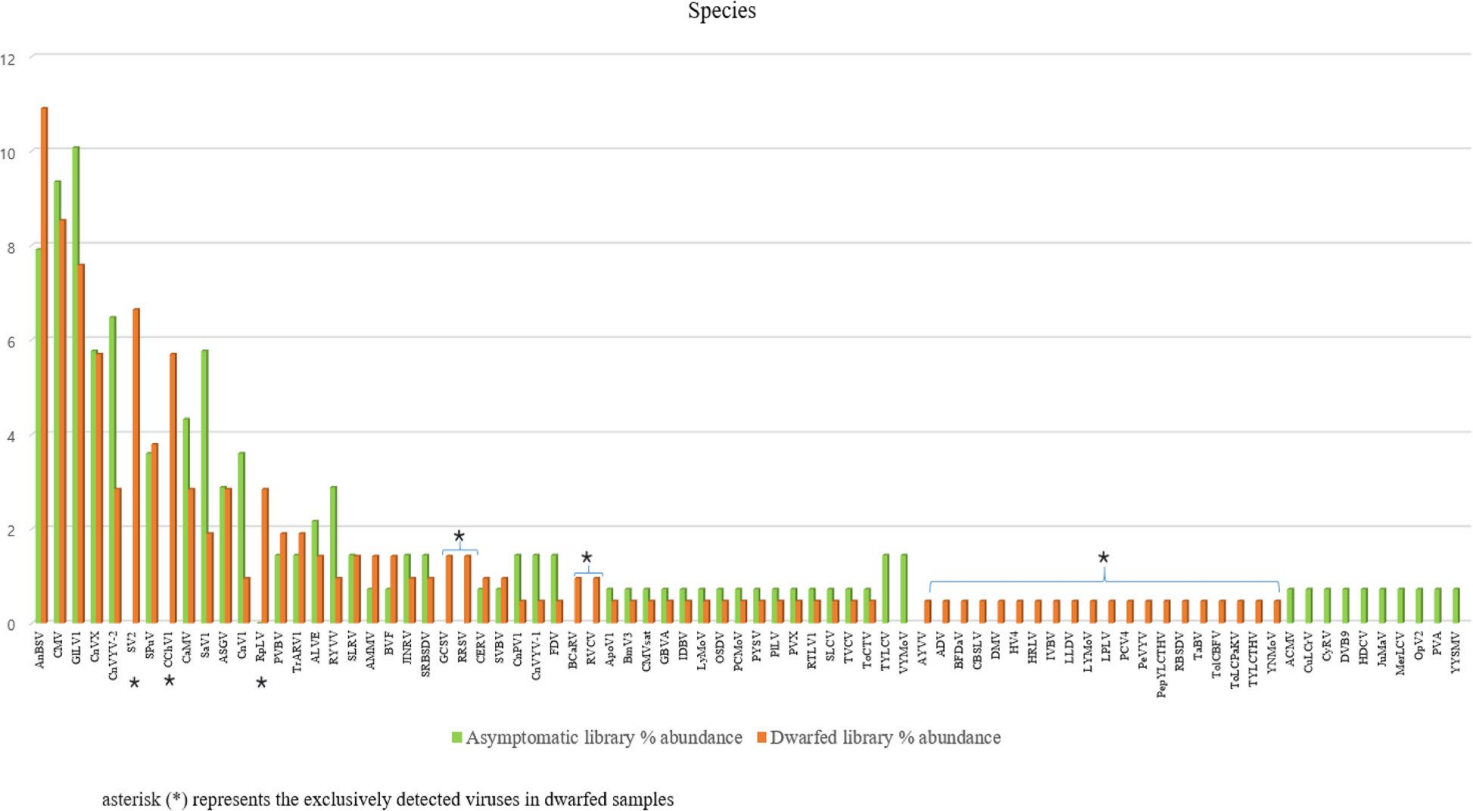
The analysis involved the identification of viral species through alignment with BLAST searches conducted in 2023, along with the determination of each virus’s relative abundance

**Fig. 5:**
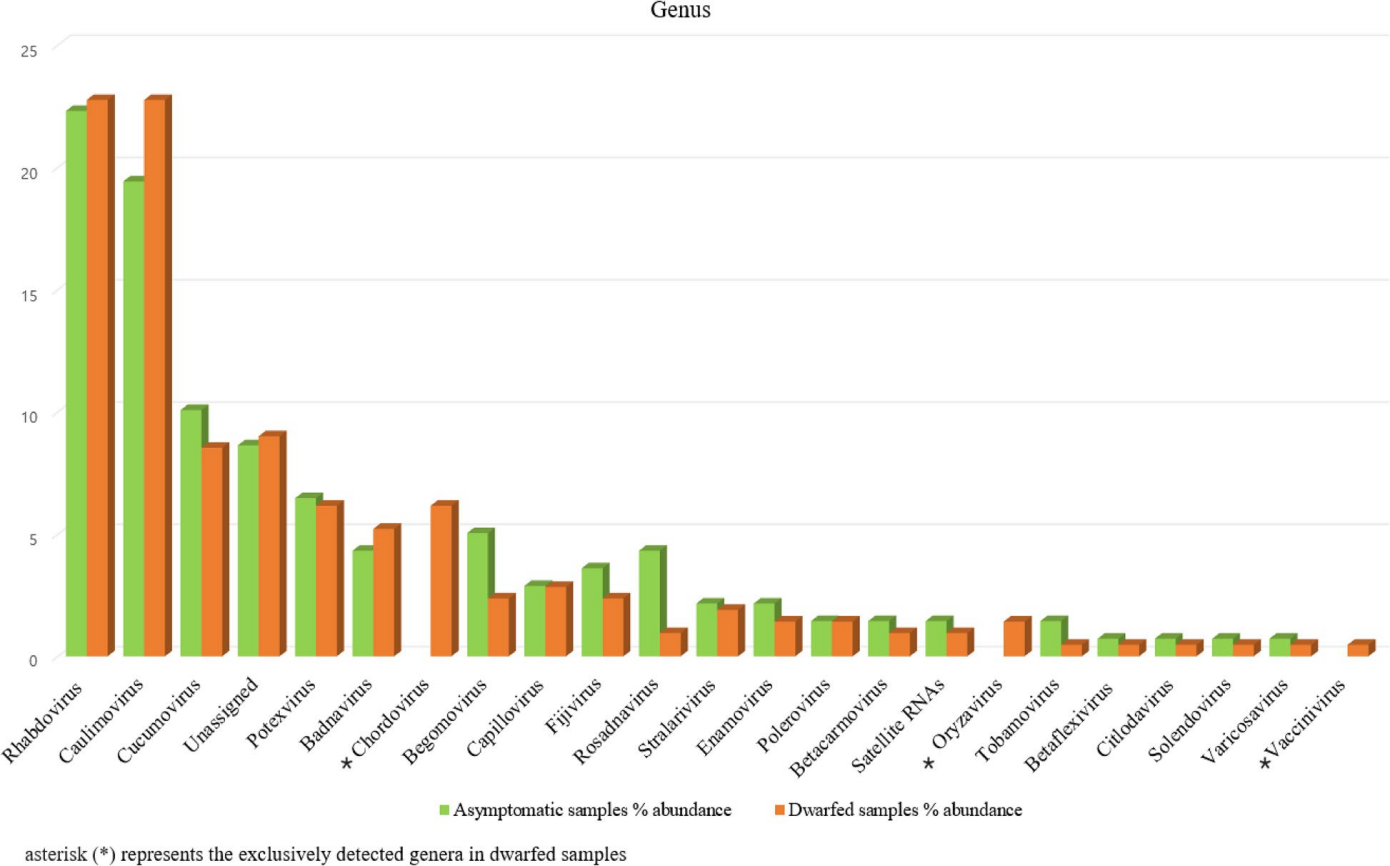
The analysis involved exploring the diversity of genera within the virome and determining the relative abundance of each genus

**Fig. 6:**
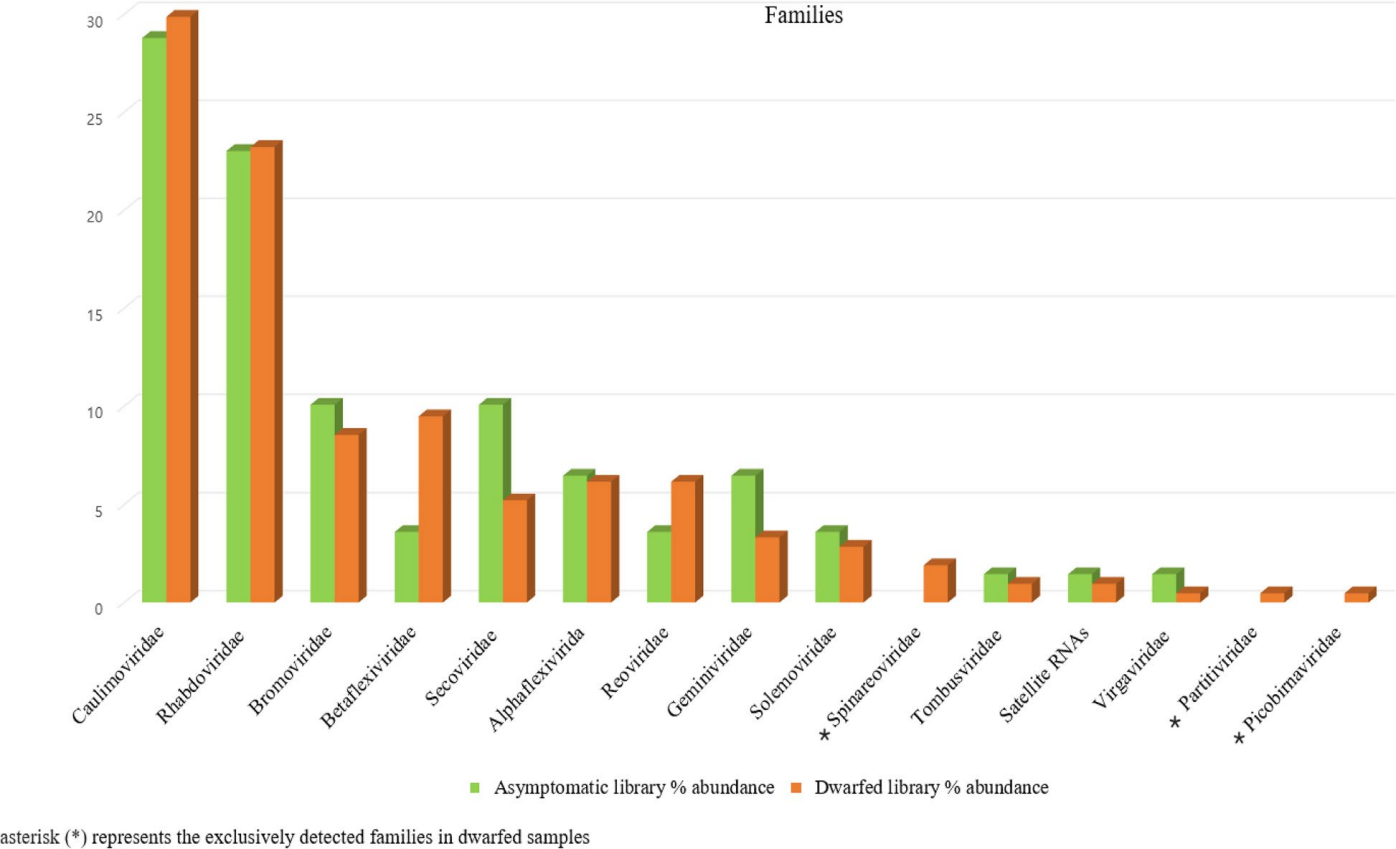
Viral family distribution within the virome of chunkugn plants in asymptomatic and dwarfed samples

A total of 350 contigs associated with 78 different viruses (Figs. 3 and 4) along with satellite RNAs were identified within the RNA-seq dataset. These viruses belong to 21 known genera and 6 unclassified genera (Fig. 5) from 14 known families (Fig. 6). Among the identified viruses, 9 were previously reported and are known pathogens of chunkung (Fig. 3). However, the remaining 69 viruses represented emerging viruses and known viruses that were not previously detected in chunkung. Additionally, some of these viruses were not previously identified in South Korea.

The selected viral contigs for the A library were 202–10,116 bp long, whereas those for the D library were 201–13,564 bp long. For both libraries, the sequence identities of the contigs varied from 21 to 100% (Additional file 1 Table S1). We examined the distribution of the detected plant viruses within each library by analyzing the relative abundance of virus-associated contigs (Additional file 1 Table S5). The most prevalent viruses in the A library were *Glehnia littoralis virus* 1 (GlLV1; 10.07%), followed by *Cucumber mosaic virus* (CMV; 9.35%), *Angelica bushy stunt virus* (AnBSV; 7.91%), *Cnidium vein yellowing virus 2* (CnVYV-2; 6.47%), and *Cnidium virus X* (CnVX; 5.76%). The most common viruses in the D library were AnBSV (10.9%), followed by CMV (8.53%), GlLV1 (7.58%), *Strawberry virus 2* (SV2; 6.64%), CnVX (5.69%), and *Carrot Ch virus 1* (CChV-1; 5.69%) (Fig. 4, Additional file 1 Table S5). The most prevalent genera in both libraries were *Rhabdovirus* (22.57%), *Caulimovirus* (21.43%), and *Cucumovirus* (9.14%) (Fig. 5, Additional file 1 Table S6), but these contigs were more abundant in the D library than in the A library. Additionally, the genera *Chordovirus*, *Oryzavirus*, and *Vaccinivirus* were identified exclusively in the D library (Fig. 5). Furthermore, viruses in the families *Partitiviridae*, *Picobirnaviridae*, and *Spinareoviridae* were identified exclusively in the dwarfed plants (Fig. 6).

Among the 78 viruses identified on the basis of plant viral gene homology for the two HTS libraries, 25 distinct viruses were detected only in the dwarfed plants (Fig. 4). Intriguingly, a shared viral community comprising 41 viruses was identified in both libraries. We analyzed the viruses that were exclusive to the dwarfed or asymptomatic plants. In the A library, 51 viruses were identified, whereas 66 viruses were identified in the D library (Table 2).

**Table 2:** HTS-based virus detection—List of virus families, genome types, and relative percent abundance (> 201 nt), and their morphology

Genome	Family	Morphology	Genus	Asymptomatic plant			Dwarfed plant			Total		
				no. contigs	no. viruses	Abundance (%)	no. contigs	no. viruses	Abundance (%)	no. contigs	no. viruses	Abundance (%)
^**a**^circular dsDNA-RT (23virus, 103 contigs)	*Caulimoviridae*	*Icosahedral/bacilliform*	*Badnavirus*	6	5	4.32	11	6	5.21	17	8	4.86
			*Caulimovirus*	27	8	19.42	48	10	22.75	75	11	21.43
			*Rosadnavirus*	6	2	4.32	2	1	0.95	8	2	2.29
			*Solendovirus*	1	1	0.72	1	1	0.47	2	1	0.57
			*Vaccinivirus*				1	1	0.47	1	1	0.29
^**b**^circular ssDNA (12 viruses, 16 contigs)	*Geminiviridae*	*Icosahedral*	*Begomovirus*	7	6	5.04	5	5	2.37	12	10	3.43
			*Citlodavirus*	1	1	0.72	1	1	0.47	2	1	0.57
			Unassigned	1	1	0.72	1	1	0.47	2	1	0.57
^**c**^linear dsRNA (9 viruses, 24 contigs)	*Spinareoviridae*		*Fijivirus*	5	3	3.6	5	4	2.37	10	4	2.86
	*Reoviridae*		Unassigned				9	2	4.27	9	2	2.57
	*Partitiviridae*	*Icosahedral*	Unassigned				1	1	0.47	1	1	0.29
	*Picobirnaviridae*		Unassigned				1	1	0.47	1	1	0.29
	*Spinareoviridae*		*Oryzavirus*				3	1	1.42	3	1	0.86
^**d**^ssRNA (-) (13 viruses, 81 contigs)	*Rhabdoviridae*	*Bullet-shaped, helical*	*Betanucleorhabdovirus*	5	1	3.6	2	1	0.95	7	1	2
			*Cytorhabdovirus*	10	2	7.19	29	8	13.74	39	8	11.14
			Unassigned	16	3	11.51	17	2	8.06	33	3	9.43
			*Varicosavirus*	1	1	0.72	1	1	0.47	2	1	0.57
^**e**^ssRNA( +) (21 viruses, 122 contigs)	*Tombusviridae*	*Icosahedral*	*Betacarmovirus*	2	1	1.44	2	1	0.95	4	1	1.14
	*Betaflexiviridae*	*Filamentous, flexible*	*Betaflexivirus*	1	1	0.72	1	1	0.47	2	1	0.57
			*Capillovirus*	4	1	2.88	6	1	2.84	10	1	2.86
			*Chordovirus*				13	2	6.16	13	2	3.71
	*Bromoviridae*	*Icosahedral/bacilliform*	*Cucumovirus*	14	2	10.07	18	1	8.53	32	2	9.14
	*Solemoviridae*	*Icosahedral*	*Enamovirus*	3	1	2.16	3	1	1.42	6	1	1.71
			*Polerovirus*	2	1	1.44	3	3	1.42	5	3	1.43
	*Alphaflexivirida*	*Filamentous, flexible*	*Potexvirus*	9	2	6.47	13	2	6.16	22	2	6.29
	*Unclassified*		Satellite RNAs	2	2	1.44	2	2	0.95	4	2	1.14
	*Secoviridae*	*Icosahedral*	*Stralarivirus*	3	2	2.16	4	2	1.9	7	2	2
			Unassigned	11	2	7.91	7	2	3.32	18	2	5.14
	Virgaviridae	*Filamentous, rigid/rod-shaped*	*Tobamovirus*	2	2	1.44	1	1	0.47	3	2	0.86
Total				139	51		211	66		350	78	

^**a**^Reverse transcribing circular double stranded DNA

^**b**^circular single stranded DNA

^**c**^linear double stranded RNA

^**d**^single-stranded RNA with a negative sense

^**e**^single-stranded RNA with a positive sense. The table data describe the number of viruses detected in each library along with their respective contig counts

### Genome composition and relative abundance of detected viruses in chunkung

**Fig. 7:**
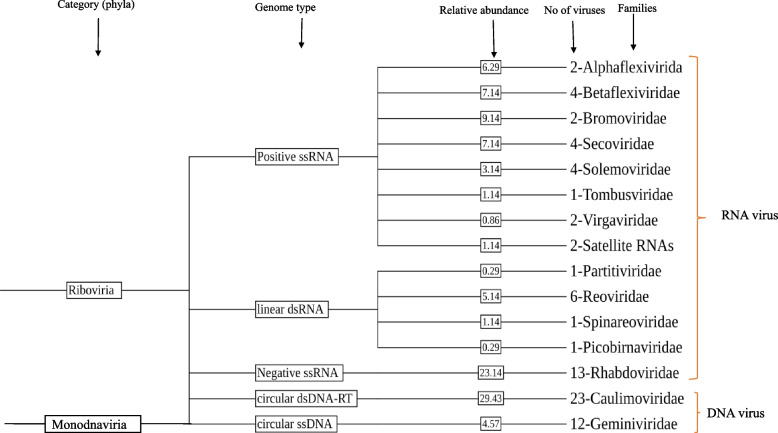
The Baltimore classification of viruses in the chunkung plant

During our analysis of the RNA-seq data, we determined the relative abundance of each virus according to its genome type and family (Fig. 7). The viromes included the circular double-stranded DNA with reverse transcriptase (dsDNA-RT) genome (n = 23; 29.43%) belonging to the family *Caulimoviridae*, the circular single-stranded DNA (ssDNA) genome (n = 12; 4.57%) belonging to the family *Geminiviridae*, the linear double-stranded RNA (dsRNA) genome (n = 9; 6.86%) belonging to the families *Reoviridae*, *Partitiviridae*, *Picobirnaviridae*, and *Spinareoviridae*, the positive-sense, single-stranded [ssRNA( +)] genome (n = 21; 36%) belonging to the families *Tombusviridae*, *Betaflexiviridae*, *Alphaflexiviridae*, *Bromoviridae*, *Solemoviridae*, *Secoviridae*, and *Virgaviridae* as well as satellite RNAs, and the negative-sense, single-stranded RNA [ssRNA( −)] genome (n = 13; 23.14%) from the family *Rhabdoviridae*. These findings provide valuable insights into the virome composition of this plant species. The RNA virus:DNA virus ratio (43:35) reflected the greater prevalence of RNA viruses in the chunkung virome.

### Confirmation of the viruses identified by HTS via (RT-) PCR assay

**Fig. 8:**
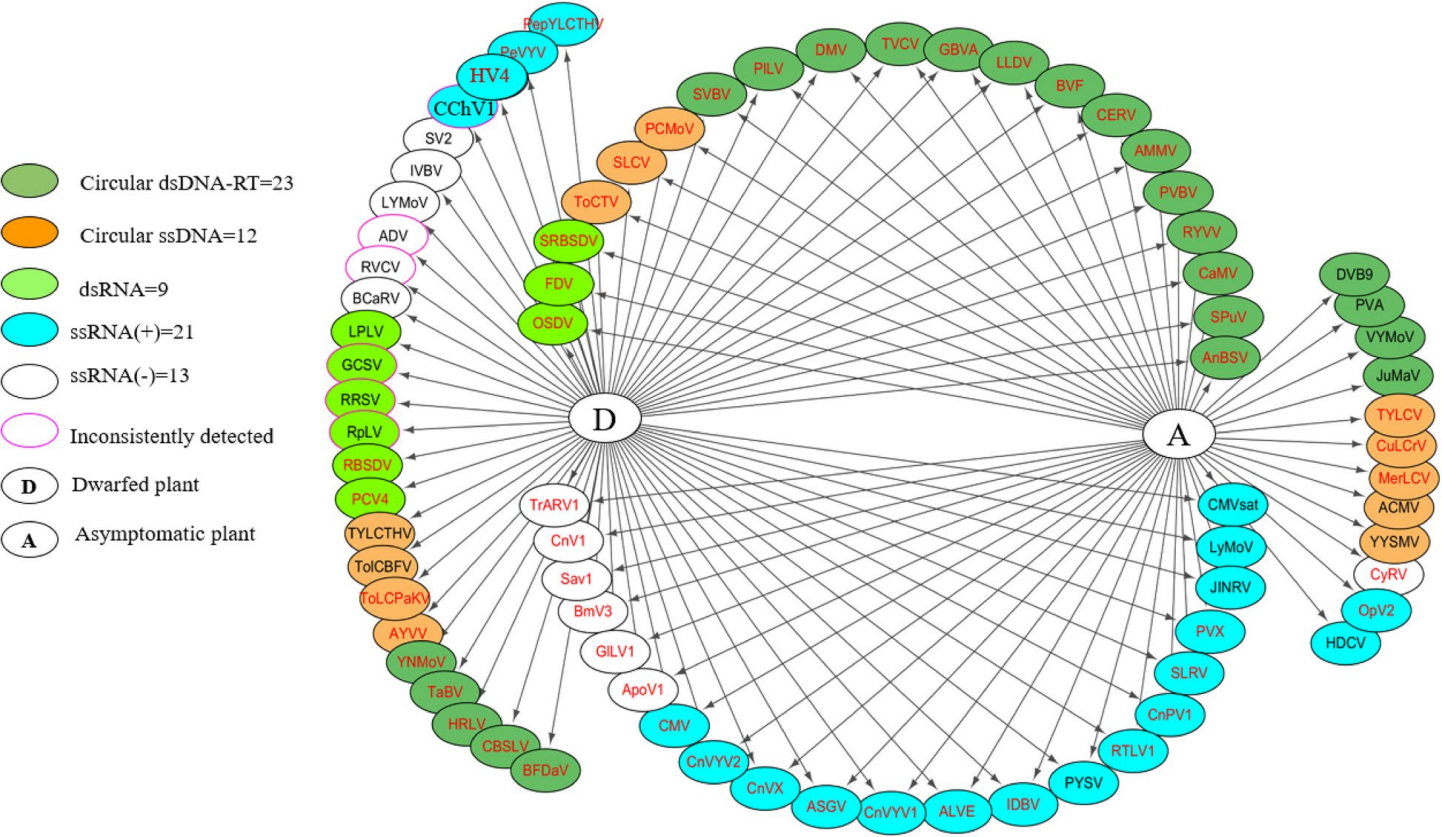
A tripartite network illustrating the overlap of viruses in chunkung samples. The network is divided into three sections: dwarfed-associated viruses (left side), asymptomatic latent-associated viruses (right side), and a shared viral community (center). The viruses are represented as nodes and are color-coded according to their genome type. Furthermore, viruses confirmed via (RT-) PCR are displayed in red font, while unconfirmed viruses are shown in black font. The acronyms for all the viruses are described in Table 3. NB: The PlLV and SLRV contigs were found in both dwarfed and asymptomatic plants in the HTS dataset, but their presence was confirmed exclusively in dwarfed plants via RT-PCR detection (Fig. 8)

The viruses identified following the analysis of the HTS data were verified via RT-PCR for RNA viruses and PCR for DNA viruses in chunkugn plants (Fig. 8) using 73 primer pairs (Additional file 2 Table S8) and a specific thermocycling program (Additional file 2 Table S9). Here, we employ three network metrics (tripartite network analysis) to illustrate the distribution of virome genome types within the chunkugn plant, revealing the intricate relationships among various viruses and their effects on the plant health. These distributions were categorized into asymptomatic (on the right side), putative dwarf plant-associated (on the left side), and shared viral communities (in the center). Subsequently, their presence was confirmed via reverse- transcriptase (RT-) PCR (Fig. 8). The result showed that although six viruses were inconsistently confirmed as present in the dwarfed samples, 12 viruses were detected in all dwarfed samples. In contrast, 5 viruses were detected only in the asymptomatic samples (Fig. 8). According to the HTS datasets, *Lamium leaf distortion virus* (LLDV) and *Dahlia mosaic virus* (DMV) were detected exclusively in the dwarfed plants. Interestingly, the PCR analysis indicated these two viruses were also present in the asymptomatic samples, indicative of the importance of employing complementary detection methods [19, 53, 54] for the comprehensive identification of viruses. All viral genomes were confirmed by (RT-) PCR, including dsDNA (19 viruses), ssDNA (8 viruses), dsRNA (8 viruses) and ssRNA (26 viruses) (Fig. 8).

#### Double-stranded reverse transcriptase DNA viruses

Of the 23 dsDNA-RT viruses identified by HTS, 19 were confirmed via PCR assays. These confirmed viruses belong to the genera *Badnavirus* (6 viruses), *Caulimovirus* (10 viruses), *Rosadnavirus* (1 virus), *Solendovirus* (1 virus), and *Vaccinivirus* (1 virus), in comparison to the total viruses detected by HTS (Table 3).

**Table 3:** A list of viruses identified based on RNA sequencing data and their PCR/RT-PCR validation in the chunkugn samples collected from production fields in south Korea in 2021; where Bold font: viruses not confirmed via PCR/RT-PCR assay and viruses indicated with (^a^): viruses known to infect chunkugn plant

Genome type	Family	Genus	Virus name	Abbreviation	Total reads	Abundance (%)	Identity (%)	Contig length
circular dsDNA,RT	Caulimoviridae	Badnavirus	Blackberry virus F	BVF	69	1.14	50–76	204–472
			Cacao Bacilliform SriLanka Virus	CBSLV	12	0.29	46	327
			Gulupa bacilliform virus A	GBVA	51	0.57	68–77	264–266
			**Jujube mosaic-associated virus**	JuMaV	59	0.29	41	569
			Pelargonium vein banding virus	PVBV	142	1.71	45–85	249–1051
			Taro bacilliform virus	TaBV	40	0.29	57	492
			Yacon necrotic mottle virus	YNMoV	17	0.29	79	341
			**Dioscorea bacilliform TR virus**	DVB9	42	0.29	81	408
		Caulimovirus	Angelica bushy stunt virus^a^	AnBSV	22,898	9.71	40–100	201–8323
			Atractylodes mild mottle virus	AMMV	91	1.14	37–61	303–475
			Carnation etched ring virus	CERV	48	0.86	53–80	242–645
			Cauliflower mosaic virus	CaMV	1316	3.43	35–72	206–1518
			Plantago latent caulimovirus	PlLV	129	0.57	47–82	242–798
			**Pueraria virus A**	PVA	158	0.29	41	897
			Soybean Putnam virus	SPuV	6949	3.71	37–88	222–4275
			Strawberry vein banding virus	SVBV	436	0.86	41–66	445–1390
			Dahlia mosaic virus	DMV	10	0.29	72	272
			Horseradish latent virus	HRLV	7	0.29	42	209
			Lamium leaf distortion virus	LLDV	220	0.29	50	975
		Rosadnavirus	Rose yellow vein virus	RYVV	559	1.71	43–59	240–673
			**Viola yellow mottle virus**	VYMoV	95	0.57	50–56	293–322
		Solendovirus	Tobacco vein clearing virus	TVCV	11	0.57	37–47	203–439
		Vaccinivirus	Blueberry fruit drop associated virus	BFDaV	19	0.29	64	341
circular ssDNA	Geminiviridae	Begomovirus	**African cassava mosaic virus**	ACMV	76	0.29	50	456
			Cucurbit leaf crumple virus	CuLCrV	12	0.29	45	209
			Merremia leaf curl virus	MerLCV	3	0.29	60	220
			Squash leaf curl virus	SLCV	50	0.57	65–82	269–289
			**Tomato leaf curl Burkina Faso virus**	TolCBFV	11	0.29	50	255
			Tomato leaf curl Pakistan virus	ToLCPaKV	22	0.29	54	243
			**Tomato yellow leaf curl Thailand virus**	TYLCTHV	82	0.29	48	852
			Tomato yellow leaf curl virus	TYLCV	33	0.57	51–52	202–43
			**Yam yellow spot mosaic virus**	YYSMV	61	0.29	49	338
			Ageratum yellow vein virus	AYVV	1	0.29	54	219
		Citlodavirus	Passion fruit chlorotic mottle virus	PCMoV	1515	0.57	44–45	2056–2978
		Unassigned	Tomato curly top virus	ToCTV	527	0.57	57–59	659–1490
linear dsRNA	Reoviridae	Fijivirus	Fiji disease virus	FDV	205,206	0.86	22–32	3759–4564
			Oat sterile dwarf virus	OSDV	104,808	0.57	22–23	1975–2018
			Rice black streaked dwarf virus	RBSDV	95,274	0.29	32	4570
			Southern rice black-streaked dwarf virus	SRBSDV	323,936	1.14	21–23	3576–3610
		Unassigned	Grapevine Cabernet Sauvignon reovirus	GCSV	414	0.86	22–36	794–1386
			Raspberry latent virus	RpLV	479	1.71	27–53	401–1738
	Partitiviridae	Unassigned	Panax cryptic virus 4	PCV4	6	0.29	52	262
	Picobirnaviridae	Unassigned	**Lysoka partiti-like virus**	LPLV	9	0.29	54	222
	Spinareoviridae	Oryzavirus	Rice ragged stunt virus	RRSV	34	0.86	39–46	317–386
ssRNA (-)	Rhabdoviridae	Betanucleorhabdovirus	Cnidium virus 1^a^	CnV1	67,823	2	77–100	779–8725
		Cytorhabdovirus	**Ivy vein banding virus**	IVBV	15	0.29	90	285
			Black currant cytorhabdovirus 1	BCaRV	45	0.57	58–60	239–492
			**Lettuce yellow mottle virus**	LYMoV	30	0.29	60	426
			Raspberry vein chlorosis virus	RVCV	21	0.57	58–64	225–237
			**Strawberry virus 2**	SV2	406	4	51–89	204–998
			Alfalfa dwarf virus	ADV	3	0.29	51	218
			Sambucus virus1	SaV1	50,191	3.43	60–92	258–13,564
			Trichosanthes associated rhabdovirus 1	TrARV1	3384	1.71	63–91	275–3010
		Unassigned	Bacopa monnieri virus 3	BmV3	125	0.57	36–40	539–610
			Cynara cardunculus rhabdovirus	CyRV	10	0.29	93	248
			Glehnia littoralis virus 1	GlLV1	4697	8.57	48–98	205–2498
		Varicosavirus	Aponogeton virus 1	ApoV1	3903	0.57	40–74	1789–1829
ssRNA( +)	Tombusviridae	Betacarmovirus	**Japanese iris necrotic ring virus**	JINRV	12	1.14	88–95	214–421
	Betaflexiviridae	Betaflexivirus	Iris domestica betaflexivirus 1	IDBV	47,633	0.57	85–86	5023–5024
		Capillovirus	Apple stem grooving virus^a^	ASGV	76,642	2.86	95–100	243–6507
		Chordovirus	Carrot Ch virus 1	CChV1	760	3.43	60–95	232–1879
			Hogweed virus 4	HV4	15	0.29	88	591
	Bromoviridae	Cucumovirus	Cucumber mosaic virus^a^	CMV	17,428,208	8.86	87–100	205–3380
			**Hydrocharis dubia cucumovirus**	HDCV	159	0.29	97	422
	Solemoviridae	Enamovirus	Arracacha latent virus E associated RNA	ALVE	4,238,694	1.71	49–85	1078–2923
		Polerovirus	Cnidium polerovirus 1^a^	CnPV1	578,766	0.57	96–98	343–6094
			Pepper vein yellows virus	PeVYV	40	0.29	94	547
			Pepper yellow leaf curl Thailand virus	PepYLCTHV	12	0.29	67	238
	Alphaflexivirida	Potexvirus	Cnidium virus X^a^	CnVX	120,246	5.71	79–100	204–5422
			Potato virus X	PVX	43,104	0.57	33	2127–2155
	Unclassified	Satellite RNAs	**Cucumber mosaic virus satellite RNA**	CMVsat	7553	0.57	93–97	363–711
			**Peony yellowing associated secovirus satellite RNA**	PYSV	555,062	0.57	67	1016–1017
	Secoviridae	Stralarivirus	**Lychnis mottle virus**^**a**^	LyMoV	289,682	0.57	96–97	3685–3710
			Strawberry latent ringspot virus	SLRV	5289	1.43	82–98	394–517
		Unassigned	Cnidium vein yellowing virus 1^a^	CnVYV1	350,522	0.86	91–100	1476–6914
			Cnidium vein yellowing virus 2^a^	CnVYV2	143,789	4.29	94–100	216–2530
	Virgaviridae	Tobamovirus	Opuntia virus 2	OpV2	97	0.29	85	672
			Rubber tree latent virus 1	RTLV1	43,403	0.57	36	8256–8315

#### Single-stranded DNA viruses

Of the 12 ssDNA viruses identified by HTS, 8 were confirmed via PCR assays. These confirmed viruses belong to the genus *Begomovirus* (n = 6 viruses) as compared to the 10 viruses detected by HTS. Viruses belong to the genus *Citlodavirus* and the *unclassified Geminiviridae* were also confirmed by PCR (Table 3).

#### Double-stranded RNA viruses

Of the 9 dsRNA viruses identified by HTS, 8 were confirmed by RT-PCR. All HTS-identified viruses belong to the genus *Fijivirus* were confirmed by RT-PCR. Additionally, two unclassified viruses in the family *Reoviridae*, one unclassified virus in the family *Partitiviridae*, and 1 virus in the genus *Oryzavirus* were also confirmed by RT-PCR (Table 3).

#### Positive-sense, single-stranded viruses

Of the 21 ssRNA( +) viruses identified by HTS, 16 were confirmed by RT-PCR assays. RT-PCR analyses confirmed the presence of these viruses from 10 known genera namely, *Betacarmovirus, Betaflexivirus, Capillovirus, Chordovirus, Cucumovirus, Enamovirus, Polerovirus, Potexvirus, Stralarivirus and Tobamovirus* (Table 3).

#### Negative-sense, single-stranded viruses

Of the 13 ssRNA( −) viruses identified by HTS, 10 were confirmed by RT-PCR assays. RT-PCR analyses confirmed the presence of 10 viruses in the *Rhabdoviridae* family compared to the number of viruses detected by HTS (Table 3).

#### Common chunkugn viruses

Angelica bushy stunt virus (AnBSV), cnidium polerovirus 1 (CnPV1), cnidium vein yellowing virus 1 and 2 (CnVYV-1 and 2), cnidium virus 1 (CnV1), cnidium virus X (CnVX), apple stem grooving virus (ASGV), and cucumber mosaic virus (CMV). These viruses were detected in all the samples as described in Table 3.

#### Distribution of viruses in dwarfed chunkung plants

Our analysis indicated that compared with the asymptomatic plants, the dwarfed plants were infected with more viruses. 12 viruses (7 DNA viruses and 5 RNA viruses) were consistently detected in all dwarfed plants.

**Fig. 9:**
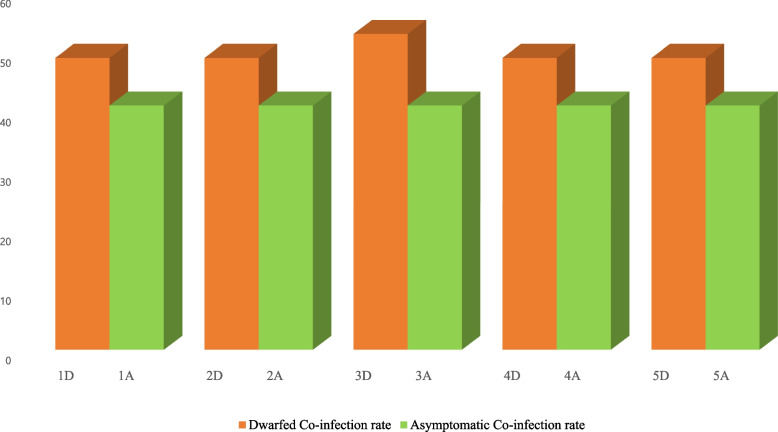
The analysis of the total number of viruses infected in each chunkugn sample was determined via (RT-) PCR detection

In terms of the total number of presumed viruses associated with dwarfed and asymptomatic plants based on genome type, and their relative abundance associated with dwarfed and asymptomatic plants, ssRNA( +) viruses were the predominant group in both asymptomatic and dwarfed plants, followed by circular dsDNA-RT viruses. We also examined the infection rates of each sample by performing (RT-) PCR analysis (Fig. 9). Except for the third sample (3D), all samples had uniform infection rates, with an equal number of viruses. The asymptomatic samples were infected with 41 viruses, which was fewer than the 49 viruses infecting the dwarfed samples. A total of 53 viruses were detected in sample 3D.

## Discussion

The advancement of virome databases, powered by reliable and precise HTS techniques, has transformed virology and created new avenues for research opportunities. These include managing disease risks [54], tracking emerging diseases, and implementing new phytosanitary regulations [55]. Hence, HTS offers a significant advantage for virome characterization over conventional techniques such as ELISA, microarray, or (RT-) PCR detection because its efficacy is primarily dictated by the completeness of the reference databases [56], the depth of the data [57], and the accuracy of the base calls [58]. By analyzing viral genomes at a large scale [59], researchers can gain insights into factors such as transmission routes and epidemiology, host range, genetic diversity [6], and evolutionary dynamics [60].

**Fig. 10:**
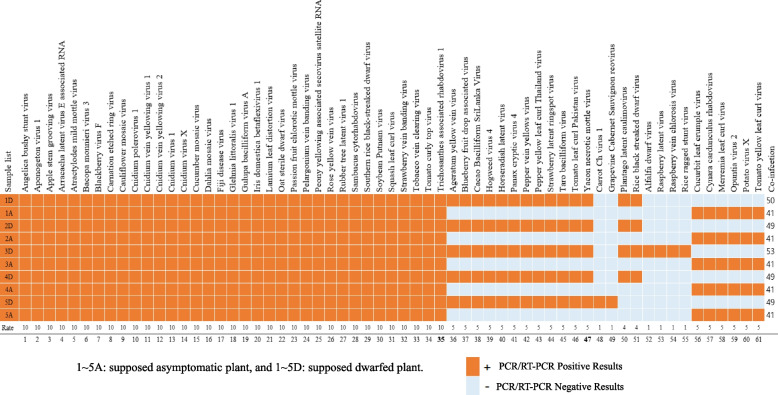
The virome composition of chunkung plants and its validation via (RT-) PCR. It also depicts the co-infection status of each sample

In this study, we used HTS analyses to identify viral populations and their genome types in asymptomatic and dwarfed chunkugn plants (Fig. 1), which appear to be resistant and susceptible (Fig. 1) to the various viruses, respectively. Although chunkugn plants are susceptible to a wide range of RNA viruses, it was surprising that different DNA and RNA viruses could co-infect chunkugn plants. Here, we discuss the efficacy of HTS techniques in revealing a wide range of biologically significant breakthroughs. Due to its broad-spectrum approach, universality, and accurate pathogen profiling, HTS is capable of effectively detecting multiple viruses, both known and novel [61], in infected samples, even at minimal concentrations [56]. This holistic approach significantly reduces the risk of false negatives, enabling us to identify various co-infections (Fig. 10) and explore the untapped potential of viral diversity in chunkugn plants. In total, based on sequencing reads from 78 HTS-detected viruses, 61 viruses were collectively confirmed via (RT-) PCR in either of the collected samples (1D, 1A ~ 5D, 5A), as shown in Fig. 10. More specifically, the analysis confirmed the presence of 35 viruses in each sample, which is indicative of the multiple co-infections of individual plants. These viruses are broadly distributed and can infect and putatively persist in chunkung plants, regardless of the plant’s health status. According to our analysis, the viruses belong to no fewer than 21 genera (Fig. 5), representing 14 different families (Fig. 7). The strength of HTS techniques lies in their ability to identify potential chunkugn viruses (Fig. 10, Table 3), particularly in cases where disease symptoms are absent, ambiguous, or attributed to only a limited number of viruses, which may not be detectable using conventional methods. This capability is particularly crucial in the context of viral diseases, where early detection plays a pivotal role in implementing effective control strategies. In previous studies, researchers have investigated viruses infecting chunkugn plants [9, 28]. However, comprehensive studies on the chunkugn virome have been limited [3, 4, 9, 29], and there were no reports of DNA viruses. Our study filled this gap and identified the co-infection of chunkugn by four different virus genome types (i.e., ssRNA, dsRNA, ssDNA, and dsDNA) (Fig. 8). The interaction of different viruses can exacerbate disease symptoms in chunkugn plants and potentially spread them, indicating the possibility of further biodiversity and quasispecies development (different genome sequences within the same host) due to their rapid replication and mutation rates [38]. Therefore, chunkugn plant can serve as good model for investigating the co-infection caused by diverse viruses and their genetic variants.

According to this study, our analysis of the distribution of DNA and RNA viruses in chunkung plants indicated that RNA viruses were more abundant than DNA viruses (Additional file 2, Table S11). This prevalence can be explained by several factors [49]. First, compared with DNA viruses, RNA viruses have higher mutation rates [20], allowing them to rapidly adapt to changing conditions and infect plants. Second, RNA viruses have smaller genomes [37] that can be efficiently packaged and replicated within plant cells. Additionally, because of their relatively small genomes, RNA viruses are easily transmitted between plants and vectors [49]. Third, diverse genetic variants may be generated in RNA viruses via recombination [11, 62] and reassortment [11, 63]. Fourth, RNA viruses rely on the host plant cellular machinery for replication and protein synthesis [11], allowing them to establish infections more efficiently than DNA viruses. Finally, the mechanisms mediating the transmission of RNA viruses (e.g., insect vectors or pollen) enhance their dispersal among plant populations [24]. However, the occurrence and distribution of DNA viruses in plants can also be influenced by several factors. For instance, pararetroviruses (PRVs, family *Caulimovirida*) have wide geographical dissemination and invade a wide range of dicotyledonous and monocotyledonous plant species [64]. PRV infections can be both asymptomatic and symptomatic, and they can remain dormant in their host for an indefinite period, becoming active when confronted with specific stress [64].

In general, viruses are obligate parasites that tend to build symbiotic relationships with their hosts [65], creating a suitable environment for the co-existence of multiple viruses within a single plant. This co-infection of different viruses within the host could cause harm [38]. For instance, studies have shown that mixed infections in plants can lead to synergistic interactions [11], resulting in increased disease symptoms. Although viruses are considered primary pathogens, some may serve beneficial roles as symbiotic partners within the host plant [65]. In contrast, certain co-infected viruses may not be associated with host disease symptoms.

Despite its advantages, the HTS has limitations. For instance, while HTS analysis exclusively detected two viruses, LLDV and DMV (Fig. 10), in dwarfed plants, PCR data revealed that both viruses were also present in the asymptomatic plants. This discrepancy suggests that HTS alone may be insufficient for a thorough and accurate investigation of the complete virome [16, 41]. Furthermore, distinguishing between true viral sequences and background noise or contaminants introduced during sample preparation or sequencing is challenging [16, 66]. Future advancements may focus on reducing the cost and complexity of HTS workflows, increasing the speed and throughput of sequencing platforms, and improving bioinformatics tools for data analysis and interpretation. The evidence presented in this work can offer valuable insights for researchers developing control and preventive strategies [53], including the implementation of biosecurity protocols to curb the spread of plant viruses in the field. The significant value of HTS and bioinformatics in plant health management will bolster their effectiveness in combating viral diseases, promoting sustainable agriculture, and ensuring reliable virus diagnosis for both known and unknown viruses.

## Conclusions

The data presented herein provide evidence that chunkung serves as an important reservoir of diverse plant viruses, which may be related to its limited genetic diversity. Results revealed an unexpectedly high co-infection rate. This raises intriguing questions regarding the mechanisms underlying the co-existence of several dozen viruses within individual plants. Additional research is needed to clarify how several dozen viruses can co-exist and interact with each other in a single plant. This study generated essential data for predicting viral outbreaks and for managing the potential risks related to the spread of phytopathogenic viruses in agroecological systems.

### Supplementary Information

**Additional file 1: Supplementary** **Table** **1.** List of BLAST annotation results for the identification of plant-associated viruses. **Supplementary** **Table** **2**. Summary of the raw and trimmed data for individual libraries sequenced on the HiSeq 4000 platform. **Supplementary** **Table** **3.** Summary sequencing data, overall mapping ratios, and annotation results for each library. **Supplementary** **Table** **4.** Statistical summary of the trinity *de novo* transcriptome assembly. Raw sequences read in each library. **Supplementary** **Table** **5.** List of identified plant viral annotations of each library. **Supplementary** **Table** **6.** List of genera identified in each library. **Supplementary** **Table** **7.** List of identified families in each library.

**Additional** **file** **2:** **Supplementary** **Table** **8.** Primer pairs used for the detection and confirmation of the HTS-detected viruses of chunkung***.*** **Supplementary** **Table** **9.** **(**RT‒) PCR thermocycling conditions used for the detection of viruses in associated chunkung. **Supplementary** **Table** **10.** Number of viruses detected in each C. officinale library, categorized by genome type**.** **Supplementary** **Table 11.** The number of detected contigs in each *Cnidium officinale* library is categorized by genome type. **Supplementary** **Table** **12.** The rates of co-infection observed in each sample, including both dwarfed and healthy plants.

## Data Availability

No datasets were generated or analysed during the current study.
